# Combined fibrinogen concentration and neutrophil-to-lymphocyte ratio, an integrative model of the inflammatory response and coagulation cascades, for predicting prognosis in patients with upper tract urothelial carcinoma

**DOI:** 10.17305/bb.2024.11039

**Published:** 2024-11-08

**Authors:** Yangqin Zheng, Chen Chen, Chaoyue Lu, Yongxing Bao, Weishi Zhang, Haote Liang, Tingyu Ye, Zhixian Yu, Yeping Li, Lina Zhou, Deguan Yu, Binwei Lin

**Affiliations:** 1 Department of Hematology, The Third Clinical Institute Affiliated to Wenzhou Medical University (Wenzhou People’s Hospital), Wenzhou, Zhejiang, China; 2 Department of Pharmacy, The Third Clinical Institute Affiliated to Wenzhou Medical University (Wenzhou People’s Hospital), Wenzhou, Zhejiang, China; 3 Department of Urology, Changhai Hospital, Navy Medical University, Shanghai, China; 4 The First Affiliated Hospital of Wenzhou Medical University, Wenzhou, Zhejiang, China; 5 Department of Urology, Longgang People’s Hospital, Wenzhou, Zhejiang, China; 6 Department of Urology, The First Affiliated Hospital of Wenzhou Medical University, Wenzhou, Zhejiang, China; 7 Department of Nephrology, The Third Clinical Institute Affiliated to Wenzhou Medical University (Wenzhou People’s Hospital), Wenzhou, Zhejiang, China; 8 Department of Urology, Rui’an People’s Hospital, The Third Affiliated Hospital of the Wenzhou Medical University, Wenzhou, Zhejiang, China

**Keywords:** Upper tract urothelial carcinoma, prognosis, neutrophil-to-lymphocyte ratio, fibrinogen, biomarker

## Abstract

Inflammation and coagulation cascades are closely correlated with cancer occurrence and progression. This study investigated the prognostic value of the combination of plasma fibrinogen level and neutrophil-to-lymphocyte ratio (F-NLR) in patients with upper tract urothelial carcinoma (UTUC). The predictive ability of the F-NLR for overall survival (OS), cancer-specific survival (CSS), and progression-free survival (PFS) was initially established and then further validated in patients who underwent radical nephroureterectomy (RNU) for UTUC. As a result, patients were divided into three groups following the establishment of cut-off values for the neutrophil-to-lymphocyte ratio (NLR) (≥2.53 vs <2.53) and fibrinogen (≥4.55 vs <4.55) through receiver operating characteristic (ROC) curve analysis: F-NLR score 0 (low fibrinogen and low NLR), 2 (high fibrinogen and high NLR), or 1 (remaining patients). The F-NLR score was then identified as an independent risk factor for OS, CSS, and PFS (all *P* value <0.05) by multivariate regression analysis in both the training and validation cohorts. In addition, F-NLR-based nomograms for OS, CSS, and PFS were developed and evaluated using the concordance index (C-index) and calibration curves. The integration of the F-NLR into existing nomograms improved predictive accuracy compared to the use of nomograms without the F-NLR score. This suggests that the addition of F-NLR is beneficial for enhancing the accuracy of prognosis prediction in patients with UTUC. The F-NLR score may serve as a powerful predictor for patients with UTUC.

## Introduction

Upper tract urothelial carcinoma (UTUC) is a rare disease, accounting for 5%–10% of urothelial cancers, and is associated with a poor prognosis [1]. The main risk factors for UTUC are smoking and exposure to aristolochic acid [2]. Unlike bladder cancer (BC), UTUC is often detected at an advanced stage due to its pauci-symptomatic nature, leading to high rates of invasion at diagnosis [3]. Patients with UTUC generally have twice the 5-year mortality rate of patients with BC (≥ 50% vs < 25%, respectively) [4]. Early diagnosis and risk stratification of UTUC patients are crucial for informing treatment strategies. For low-risk UTUC, kidney-sparing surgery via ureteroscopy is recommended, while high-risk patients are typically offered radical nephroureterectomy (RNU). Thus, there is a need for novel predictive tools to more accurately assess the prognosis of UTUC patients.

An increasing body of research has shown that the inflammatory microenvironment plays a pivotal role in the development of various cancers [5]. One prominent inflammation-based index, the neutrophil-to-lymphocyte ratio (NLR), has been reported as an independent risk indicator in multiple cancers, including non-small-cell lung cancer [6], breast cancer [7], and gastrointestinal cancer [8]. The prognostic value of NLR in UTUC has also been established [9, 10]. Additionally, research has linked coagulation cascades with tumor biology [11]. Fibrinogen, produced by hepatocytes, plays a key role in the coagulation process, and high fibrinogen levels have been associated with poor survival in several cancers [12–14]. Elevated pretreatment fibrinogen levels may also predict poorer outcomes in patients with UTUC [15]. Recently, the combined use of NLR and fibrinogen (F-NLR) has been explored in various cancers, with studies confirming its prognostic value [5, 16]. However, the impact of F-NLR on the prognosis of patients with UTUC has not yet been reported.

In this study, we aimed to investigate the correlation between F-NLR and clinicopathological factors and to evaluate the clinical utility of F-NLR as a novel predictive biomarker for UTUC patients after RNU.

## Materials and methods

### Study population

**Figure 1.**
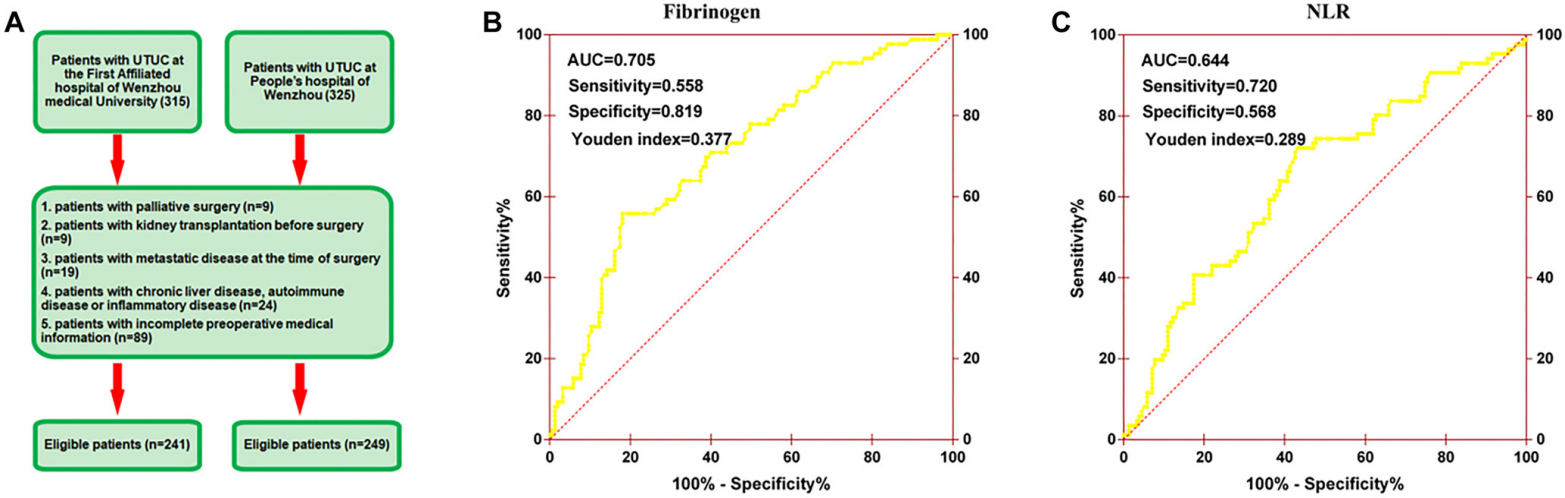
The patient selection flowchart (A) and determination of the optimal cutoff value for (B) fibrinogen and (C) NLR based on the ROC analysis. NLR: Neutrophil-to-lymphocyte ratio; ROC: Receiver operating characteristic.

A total of 640 patients who underwent RNU for UTUC at two clinical centers—The First Affiliated Hospital of Wenzhou Medical University (from March 2005 to August 2015) and the Third Clinical Institute Affiliated Hospital of Wenzhou Medical University (from July 2003 to December 2016)—were included in this study. The study flowchart, along with inclusion and exclusion criteria, is shown in Figure 1A. Overall survival (OS), cancer-specific survival (CSS), and progression-free survival (PFS) were defined as the time from surgery to the date of death from any cause, to death specifically from cancer, or to the date of radiologically or histologically confirmed tumor recurrence, respectively. This study was approved by the Ethics Committee of both centers.

### Data collection and cutoff value calculation

Patients’ clinicopathological parameters—such as gender, age, American Society of Anesthesiologists (ASA) grade, body mass index (BMI), hydronephrosis status, and other relevant factors—were retrospectively reviewed and collected. Using receiver operating characteristic (ROC) curve analysis, optimal cutoff values for the NLR and fibrinogen levels associated with OS were determined. The cutoff values were set as follows: an NLR score═0 (<2.53) and score═1 (≥2.53) (Figure 1B), and a fibrinogen score═0 (≤4.55) and score═1 (≥4.55) (Figure 1C). The F-NLR score was defined as the combined sum of the NLR and fibrinogen scores. Based on their F-NLR scores, patients were divided into three groups: Low F-NLR (score═0), Intermediate F-NLR (score═1), and High F-NLR (score═2).

### Ethical statement

This study was approved by the Medical Ethics Committee of the First Affiliated Hospital of Wenzhou Medical University (KY2023-R164) and the Third Clinical Institute Affiliated Hospital of Wenzhou (KY-2022-062), in compliance with the Declaration of Helsinki. Informed consent was waived due to the study’s retrospective nature.

### Statistical analysis

SPSS software (version 25.0) and R software (version 4.3.1) were used for statistical analyses. The Pearson chi-squared test and Student’s *t*-test were used to compare categorical and continuous variables, respectively. The impact of F-NLR on OS, CSS, and PFS was assessed using Kaplan–Meier curves and the log-rank test. Significant independent factors influencing OS, CSS, and PFS were identified through univariate and multivariate analyses (using forward selection) and subsequently incorporated into the nomogram. We tested for multicollinearity and interaction effects in the multivariate analysis and conducted model assumption tests when using Cox regression. Additionally, calibration, concordance index (c-index), and area under the curve (AUC) were used to evaluate the predictive accuracy of the nomogram. A two-sided significance was defined as *P* value<0.05

## Results

### Patient characteristics

This study enrolled a total of 490 eligible patients. Among them, 241 patients were recruited from the First Affiliated Hospital of Wenzhou Medical University and assigned to the training cohort, while 249 patients from the Third Clinical Institute Affiliated Hospital of Wenzhou comprised the validation cohort. Baseline characteristics for both cohorts, categorized by fibrinogen and NLR levels, are summarized in Table 1.

**Table 1:** Characteristics of training and validation cohorts according to fibrinogen and NLR

**Variable**	**Training cohort (*n* ═ 241)**						**Validation cohort (*n* ═ 249)**					
	**Fibrinogen**			**NLR**			**Fibrinogen**			**NLR**		
	**≥4.55** **(*n* ═ 76)**	**< 4.55** **(*n* ═ 165)**	***P* value**	**≥2.53** **(*n* ═ 129)**	**< 2.53** **(*n* ═ 112)**	***P* value**	**≥4.55** **(*n* ═ 76)**	**< 4.55** **(*n* ═ 173)**	***P* value**	**≥2.53** **(*n* ═ 131)**	**< 2.53** **(*n* ═ 118)**	***P* value**
Age, years (>65/≤65)	56/20	100/65	**0.048**	89/40	67/45	0.137	49/27	99/74	0.283	79/52	69/49	0.769
Gender (male/female)	56/20	118/47	0.727	99/30	75/37	0.091	53/23	115/58	0.613	97/34	71/47	**0.020**
ASA grade (≥3/<3)	21/55	36/129	0.324	36/93	21/91	0.095	13/63	26/147	0.678	22/109	17/101	0.605
BMI, kg/m^2^ (≥25/<25)	12/64	34/131	0.377	12/117	34/78	**<0.001**	22/54	60/113	0.375	41/90	41/77	0.563
Hydronephrosis (yes/no)	51/25	111/54	0.979	90/39	72/40	0.366	52/24	54/119	0.954	91/40	80/38	0.777
Hemoglobin, g/dL	113.53 ± 19.95	123.46 ± 21.11	**0.001**	115.95 ± 23.66	125.37 ± 16.73	**0.001**	118.91 ± 17.55	128.31 ± 18.11	**<0.001**	125.41 ± 17.64	125.52 ± 19.34	0.964
Anemia (yes/no)	48/28	55/110	**<0.001**	72/57	31/81	**<0.001**	34/42	41/132	**0.001**	46/85	29/89	0.070
Albumin, g/dL	39.27 ± 5.07	41.61 ± 4.22	**<0.001**	40.24 ± 5.24	41.60 ± 3.68	**0.023**	37.63 ± 3.62	40.06 ± 4.94	**<0.001**	38.53 ± 3.69	40.19 ± 5.52	**0.005**
Hypoproteinemia (yes/no)	13/63	8/157	**0.002**	18/111	3/109	**0.002**	14/62	8/165	**<0.001**	16/115	6/112	**0.048**
Neutrophil, 10^9^/L	6.26 ± 2.76	4.13 ± 1.55	**<0.001**	5.82 ± 2.46	3.64 ± 1.14	**<0.001**	5.76 ± 3.94	4.31 ± 2.09	**<0.001**	6.09 ± 3.29	3.26 ± 1.03	**<0.001**
Lymphocytes, 10^9^/L	1.58 ± 0.65	1.75 ± 0.66	0.062	1.39 ± 0.53	2.05 ± 0.62	**<0.001**	1.60 ± 0.72	1.71 ± 0.60	0.208	1.40 ± 0.57	1.99 ± 0.56	**<0.001**
NLR	4.66 ± 3.27	2.82 ± 2.58	**<0.001**	4.77 ± 3.44	1.82 ± 0.46	**<0.001**	3.81 ± 2.07	3.00 ± 2.59	**0.016**	4.65 ± 2.68	1.69 ± 0.48	**<0.001**
Fibrinogen, g/L	5.76 ± 1.10	3.33 ± 0.60	**<0.001**	4.53 ± 1.53	3.60 ± 1.00	**<0.001**	5.32 ± 0.80	3.20 ± 0.65	**<0.001**	4.11 ± 1.34	3.54 ± 0.96	**<0.001**
Tumor size, cm (≥3/<3)	40/36	52/113	**0.002**	55/74	37/75	0.126	33/43	54/119	0.063	54/77	33/85	**0.028**
Tumor site (pelvicalyceal/ ureter/both)	50/21/5	103/57/5	0.295	84/37/8	69/41/2	0.129	45/23/8	92/74/7	**0.046**	70/51/10	67/46/5	0.518
Multifocality (yes/no)	16/60	32/133	0.764	32/97	16/96	**0.041**	22/54	36/137	0.162	30/101	28/90	0.877
Pathologic T stage (T1/T2/T3/T4)	17/14/22/23	52/70/38/5	**<0.001**	27/42/38/22	42/42/22/6	**0.001**	11/14/40/11	72/47/46/8	**<0.001**	35/29/52/15	48/32/34/4	**0.009**
N stage (N1/N0)	18/58	5/160	**<0.001**	19/110	4/108	**0.003**	10/66	5/168	**0.004**	13/118	2/13	**0.006**
High tumor grade (yes/no)	64/12	122/43	0.078	102/27	84/28	0.453	70/6	113/60	**<0.001**	105/26	78/40	**0.012**
LVI (yes/no)	24/52	14/151	**<0.001**	28/101	10/102	**0.007**	19/57	157/16	**0.001**	27/104	8/110	**0.002**
All-cause death, *n* (%)	48 (63.16%)	38 (23.03%)	**<0.001**	62 (48.06%)	24 (21.43%)	**<0.001**	37 (48.68%)	38 (21.97%)	**<0.001**	53 (40.46%)	22 (18.64%)	**<0.001**
Cancer-specific mortality, *n* (%)	42 (55.26%)	26 (15.76%)	**<0.001**	47 (36.43%)	21 (18.75%)	**0.002**	31 (40.79%)	27 (15.61%)	**<0.001**	43 (32.82%)	15 (12.71%)	**<0.001**
Recurrence, *n* (%)	46 (60.52%)	48 (29.09%)	**<0.001**	58 (44.96%)	36 (32.14%)	**0.042**	37 (48.68%)	43 (24.86%)	**<0.001**	53 (40.46%)	27 (22.88%)	**0.003**
Follow up duration, months, median (quartile)	17.60 (10.15--40.13)	39.70 (24.30--67.70)	**<0.001**	30.00 (14.10-50.20)	39.65 (19.73--69.83)	**0.004**	35.60 (14.68--58.43)	46.80 (31.25--68.45)	**0.002**	35.10 (24.20--55.90)	53.55 (35.88--69.50)	**<0.001**

NLR: Neutrophil-to-lymphocyte ratio; LVI: Lymphovascular invasion.

In the training cohort, there was a male majority, with 174 (72.2%) male patients and 67 (27.8%) female patients. The mean age was 67.7 ± 10.5 years, and 156 patients were over the age of 65. The median follow-up period was 33.7 months (interquartile range [IQR]: 16.8–63.4 months). During the entire follow-up period, 86 patients (35.7%) died, including 68 (38.2%) who died from cancer. Additionally, 94 patients (39.0%) experienced tumor recurrence after surgery.

In the validation cohort, 168 patients (67.5%) were male, and 81 patients (32.5%) were female. More than half of the patients—148 (59.4%)—were over the age of 65, with a median age of 65.9 ± 10.4 years. The median follow-up period was 44.7 months (IQR: 27.4–64.4 months). During follow-up, 75 patients (30.1%) passed away, including 58 (23.3%) who died from cancer, and 80 patients (32.1%) experienced tumor recurrence after surgery. The parameters in Tables 1 and 2 followed a normal distribution.

The optimal cutoff values for fibrinogen and NLR were 4.55 and 2.53, respectively (Table 1). The AUC for fibrinogen and NLR were 0.705 and 0.644, respectively (Figure 1B and 1C). A high fibrinogen level (≥4.55) was associated with anemia, hypoproteinemia, more advanced pathological T and N stages, and lymphovascular invasion (LVI) (all *P* value < 0.05) in both the training and validation groups. Similarly, a higher NLR was closely associated with hypoproteinemia, more advanced pathological T and N stages, and LVI (all *P* value < 0.05) in both cohorts (Table 1).

**Table 2:** Baseline characteristics of patients with non-metastatic UTUC according to F-NLR score in training cohorts

**Variables**	**F-NLR score**			
	**0 (N ═ 95)**	**1 (N ═ 87)**	**2 (N ═ 59)**	***P* value**
Age, years (>65/≤65)	56/39	55/32	45/14	0.085
Gender (male/female)	65/30	63/24	46/13	0.437
ASA grade (≥3/<3)	19/76	19/68	19/40	0.197
BMI, kg/m^2^ (≥25/<25)	29/66	10/77	7/52	**0.001**
Hydronephrosis (yes/no)	61/34	61/26	40/19	0.694
Hemoglobin, g/dL	126.24 ± 16.99	119.86 ± 23.55	111.51 ± 20.88	**<0.001**
Anemia (yes/no)	25/70	36/51	42/17	**<0.001**
Albumin, g/dL	41.76 ± 3.66	41.26 ± 4.68	38.86 ± 5.35	**<0.001**
Hypoproteinemia (yes/no)	3/92	5/82	13/46	**<0.001**
Neutrophil, 10^9^/L	3.48 ± 1.01	4.92 ± 1.66	6.77 ± 2.86	**<0.001**
Lymphocytes, 10^9^/L	2.02 ± 0.63	1.53 ± 0.59	1.40 ± 0.58	**<0.001**
NLR	1.78 ± 0.47	3.81 ± 3.21	5.41 ± 3.35	**<0.001**
Fibrinogen, g/L	3.29 ± 0.58	3.77 ± 1.06	5.88 ± 1.11	**<0.001**
Tumor size, cm (≥3/<3)	28/67	33/54	31/28	**0.016**
Tumor site (pelvicalyceal/ ureter/both)	61/34/0	50/30/7	42/14/3	**0.009**
Multifocality (yes/no)	13/82	22/65	13/46	0.132
Pathologic T stage (T1/T2/T3/T4)	38/39/16/2	18/34/28/7	13/11/16/19	**<0.001**
N stage (N1/N0)	2/93	5/82	16/43	**<0.001**
High tumor grade (yes/no)	71/24	64/23	51/8	0.147
LVI (yes/no)	8/87	8/79	22/37	**<0.001**
All-cause death, *n* (%)	14/81	34/53	38/21	**<0.001**
Cancer-specific mortality, *n* (%)	11/84	25/62	32/27	**<0.001**
Recurrence, *n* (%)	11/84	25/62	32/27	**<0.001**
Follow up duration, months, median (quartile)	50.22 ± 30.06	41.72 ± 28.93	27.65 ± 25.57	**<0.001**

UTUC: Upper tract urothelial carcinoma; F-NLR: Fibrinogen level and neutrophil-to-lymphocyte ratio; LVI: Lymphovascular invasion; NLR: Neutrophil-to-lymphocyte ratio.

### Association between F-NLR and clinicopathological variables

Patients were stratified into three groups based on F-NLR scores: low F-NLR (score ═ 0), intermediate F-NLR (score ═ 1), and high F-NLR (score ═ 2). In the training and validation cohorts, there were 95 patients (39.4%) in the low F-NLR group, 87 patients (36.1%) in the intermediate F-NLR group, and 59 patients (24.5%) in the high F-NLR group.

Significant associations were observed between F-NLR and several clinicopathological variables, including anemia, hyperproteinemia, neutrophil and lymphocyte counts, NLR, fibrinogen levels, tumor size, pathological T and N stages, LVI, all-cause mortality, cancer-specific mortality, tumor recurrence, and OS (all *P* value < 0.05) in both the training and validation groups (Table 2 and Table S1).

### Survival and cox regression analysis of F-NLR for OS, CSS, and PFS

**Figure 2.**
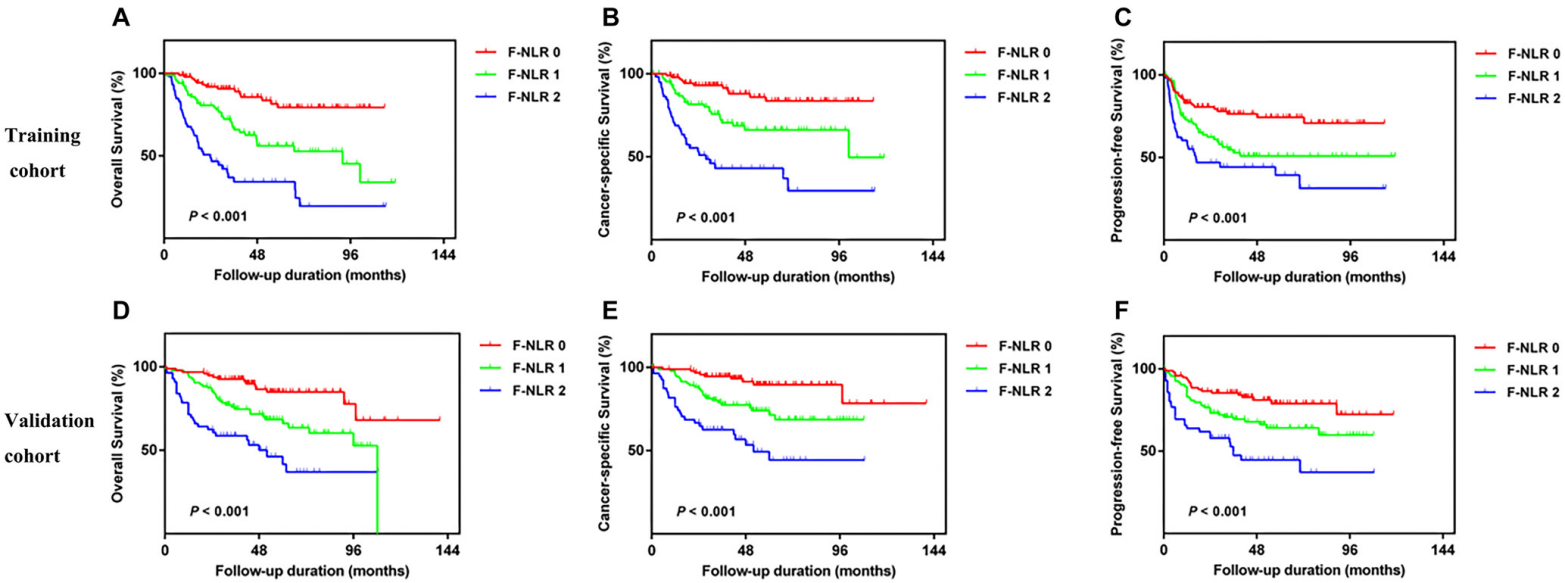
Kaplan–Meier analysis for OS, CSS, and PFS in UTUC patients according to F-NLR in the training cohort (A–C) and validation cohort (D–F). UTUC: Upper tract urothelial carcinoma; OS: Overall survival; CSS: Cancer-specific survival; PFS: Progression-free survival.

Kaplan–Meier analysis indicated that patients with an F-NLR score of 2 had significantly worse OS, CSS, and PFS compared to those with an F-NLR score of 0 or 1 (*P* value < 0.01) in both the training and validation groups (Figure 2). Univariate analysis further demonstrated that an F-NLR score of 1 or 2 had a significant impact on OS (HR ═ 3.115, 95% CI: 1.671–5.810, *P* < 0.001 for score ═ 1; HR ═ 7.442, 95% CI: 4.016–13.790, *P* < 0.001 for score ═ 2), CSS (HR ═ 2.851, 95% CI: 1.402–5.798, *P* ═ 0.004 for score ═ 1; HR ═ 7.503, 95% CI: 3.767–14.945, *P* < 0.001 for score ═ 2), and PFS (HR ═ 2.039, 95% CI: 1.214–3.425, *P* ═ 0.007 for score ═ 1; HR ═ 3.389, 95% CI: 1.985–5.787, *P* < 0.001 for score ═ 2) in the training cohort (Table 3). Additionally, other factors—such as anemia, hyperproteinemia, pathological T and N stages, LVI, and tumor grade—were also associated with OS, CSS, and PFS (all *P* value < 0.05).

**Table 3:** Univariate analysis of parameters for the prediction of survival outcomes in patients with UTUC in training cohort and validation cohort

**Parameter**	**Overall survival**			**Cancer-specific survival**			**Progression-free survival**		
	**HR**	**95% CI**	***P* value**	**HR**	**95% CI**	***P* value**	**HR**	**95% CI**	***P* value**
*Training cohort*									
Age, years (≥65 /<65)	2.174	1.324–3.569	**0.002**	2.011	1.158–3.495	**0.013**	1.735	1.103–2.731	**0.017**
Gender (male/female)	0.873	0.548–1.391	0.567	0.809	0.483–1.354	0.420	1.008	0.641–1.584	0.973
ASA grade (≥3/<3)	1.605	1.015–2.536	**0.043**	1.329	0.781–2.262	0.295	1.154	0.726–1.836	0.544
BMI, kg/m^2^ (≥25/<25)	0.404	0.195–0.837	0.015	0.389	0.168–0.901	**0.028**	0.442	0.229–0.852	**0.015**
Hydronephrosis (yes/no)	1.531	0.943–2.485	0.085	1.836	1.034–3.258	**0.038**	1.859	1.144–3.021	**0.012**
Surgical approach (laparoscopic/open)	0.632	0.383–1.043	0.072	0.680	0.393–1.178	0.169	0.696	0.440–1.100	0.121
Anemia (yes/no)	2.158	1.404–3.318	**<0.001**	1.922	1.189–3.106	**0.008**	1.925	1.281–2.894	**0.002**
Hypoalbuminemia (yes/no)	2.721	1.528–4.844	**0.001**	2.826	1.509–5.293	**0.001**	1.953	1.086–3.513	**0.025**
Tumor size, cm (≥3/<3)	1.454	0.948–2.230	0.086	1.589	0.985–2.563	0.058	1.558	1.038–2.339	**0.032**
*Tumor site*									
Pelvicalyceal	1.000	Reference	1.000	1.000	Reference	1.000	1.000	Reference	1.000
Ureter	1.240	0.786–1.956	0.356	1.426	0.859–2.366	0.170	1.474	0.960–2.264	0.076
Both	1.665	0.664–4.176	0.277	2.262	0.889–5.756	0.087	2.293	1.041–5.051	**0.039**
Multifocality (yes/no)	1.703	1.055–2.747	**0.029**	1.802	1.061–3.063	**0.029**	1.448	0.905–2.319	0.123
*Pathologic T stage*									
pT1-2	1.000	Reference	**1.000**	1.000	Reference	**1.000**	1.000	Reference	**1.000**
pT3-4	4.618	2.970–7.180	**<0.001**	5.701	3.406–9.540	**<0.001**	2.893	1.917–4.364	**<0.001**
N stage (yes/no)	7.470	4.439–12.571	**<0.001**	8.688	5.014–15.055	**<0.001**	5.016	3.032–8.298	**<0.001**
Tumor grade (≥3/<3)	2.990	1.378–6.488	**0.006**	4.418	1.607–12.145	**0.004**	2.189	1.194–4.014	**0.011**
LVI (yes/no)	5.831	3.702–9.184	**<0.001**	7.539	4.611–12.324	**<0.001**	4.302	2.766–6.692	**<0.001**
*F-NLR*									
0	1.000	Reference	**1.000**	1.000	Reference	**1.000**	1.000	Reference	**1.000**
1	3.115	1.671–5.810	**<0.001**	2.851	1.402–5.798	**0.004**	2.039	1.214–3.425	**0.007**
2	7.442	4.016–13.790	**<0.001**	7.503	3.767–14.945	**<0.001**	3.389	1.985–5.787	**<0.001**
*Validation cohort*									
Age, years (≥65 /<65)	1.248	0.772–2.019	0.366	0.944	0.557–1.598	0.829	0.962	0.615–1.504	0.864
Gender (male/female)	1.408	0.844–2.350	0.190	1.624	0.890–2.964	0.114	1.341	0.821–2.191	0.242
ASA grade (≥3/<3)	1.228	0.673–2.238	0.503	0.896	0.424–1.892	0.773	0.858	0.453–1.622	0.636
BMI, kg/m^2^ (≥25/<25)	0.788	0.475–1.309	0.358	0.784	0.441–1.395	0.408	0.937	0.583–1.504	0.787
Hydronephrosis (yes/no)	1.189	0.717–1.970	0.502	1.364	0.757–2.456	0.302	1.048	0.653–1.682	0.846
Anemia (yes/no)	2.503	1.590–3.941	**<0.001**	2.194	1.306–3.683	**0.003**	1.873	1.196–2.932	**0.006**
Hypoalbuminemia (yes/no)	1.519	0.753–3.062	0.243	1.616	0.731–3.573	0.235	1.136	0.522–2.468	0.748
Tumor size, cm (≥3/<3)	1.805	1.142–2.853	**0.011**	1.948	1.162–3.267	**0.011**	1.603	1.026–2.505	**0.038**
*Tumor site*									
Pelvicalyceal	1.000	Reference	1.000	1.000	Reference	1.000	1.000	Reference	1.000
Ureter	0.701	0.424–1.157	0.164	0.661	0.374–1.167	0.153	0.688	0.428–1.108	0.124
Both	1.896	0.889–4.043	0.098	1.428	0.559–3.650	0.457	1.003	0.399–2.520	0.994
Multifocality (yes/no)	1.476	0.898–2.429	0.125	1.358	0.763–2.417	0.298	1.091	0.652–1.825	0.741
*Pathologic T stage*									
pT1-2	1.000	Reference	1.000	1.000	Reference	1.000	1.000	Reference	1.000
pT3-4	4.997	3.018–8.273	**<0.001**	7.518	3.965–14.255	**<0.001**	4.328	2.685–6.977	**<0.001**
N stage (yes/no)	3.931	2.009–7.695	**<0.001**	3.392	1.534–7.503	**0.003**	2.515	1.209–5.235	**0.014**
Tumor grade (≥3/<3)	3.234	1.680–6.226	**<0.001**	4.037	1.842–10.072	**0.001**	2.755	1.486–5.110	**0.001**
LVI (yes/no)	2.883	1.683–4.938	**<0.001**	2.996	1.657–5.417	**<0.001**	2.622	1.560–4.405	**<0.001**
*F-NLR*									
0	1.000	Reference	1.000	1.000	Reference	1.000	1.000	Reference	1.000
1	2.590	1.381–4.857	**0.003**	2.988	1.389–6.429	**0.005**	1.915	1.085–3.379	**0.025**
2	5.367	2.829–10.182	**<0.001**	7.124	3.319–15.292	**<0.001**	3.946	2.206–7.056	**<0.001**

UTUC: Upper tract urothelial carcinoma; LVI: Lymphovascular invasion; NLR: Neutrophil-to-lymphocyte ratio.

These parameters were then included in the multivariate analysis, which showed that F-NLR and pathological T stage remained independent risk factors for OS, CSS, and PFS in the training cohort (Table 4). The results from the validation cohort similarly confirmed that F-NLR was a significant predictor of OS, CSS, and PFS (Tables 3 and 4). Finally, no multicollinearity or interaction effects were observed for these independent predictors.

**Table 4:** Multivariate analysis of parameters for the prediction of survival outcomes in patients with UTUC in training cohort and validation cohort

**Parameter**	**Overall survival**			**Cancer-specific survival**			**Progression-free survival**		
	**HR**	**95% CI**	***P* value**	**HR**	**95% CI**	***P* value**	**HR**	**95% CI**	***P* value**
*Training cohort*									
Age, years (≥65 /<65)	2.030	1.221–3.374	**0.006**	1.885	1.063–3.344	**0.030**		-	
Hydronephrosis (yes/no)				2.413	1.338–4.351	**0.003**			
Tumor site									
Pelvicalyceal		-			-		1.000	Reference	1.000
Ureter							1.985	1.271–3.099	**0.003**
Both							2.238	0.995–5.035	0.052
*Pathologic T stage*									
pT1-2	1.000	Reference	1.000	1.000	Reference	1.000	1.000	Reference	1.000
pT3-4	2.816	1.724–4.601	**<0.001**	2.995	1.645–5.454	**<0.001**	1.794	1.088–2.959	**0.022**
N stage (yes/no)	2.537	1.389–4.635	**0.002**						
Tumor grade (≥3/<3)		-			-		1.522	1.004–2.307	**0.048**
LVI (yes/no)				3.271	1.769–6.048	**<0.001**	2.863	1.643–4.991	**<0.001**
*F-NLR*									
0	1.000	Reference	1.000	1.000	Reference	1.000	1.000	Reference	1.000
1	2.401	1.270–4.540	**0.007**	2.452	1.180–5.094	**0.016**	1.701	0.989–2.926	0.055
2	3.595	1.830–7.061	**<0.001**	3.673	1.792–7.530	**<0.001**	2.016	1.118–3.634	**0.020**
*Validation cohort*									
Anemia (yes/no)	1.754	1.095–2.809	**0.019**		-			-	
*Pathologic T stage*									
pT1-2	1.000	Reference	1.000	1.000	Reference	1.000	1.000	Reference	1.000
pT3-4	3.684	2.152–6.309	**<0.001**	5.753	2.974–11.131	**<0.001**	3.606	2.194–5.927	**<0.001**
N stage (yes/no)	1.906	0.936–3.879	0.075						
*F-NLR*									
0	1.000	Reference	1.000	1.000	Reference	1.000	1.000	Reference	1.000
1	1.988	1.046–3.778	0.036	2.485	1.151–5.365	**0.020**	1.721	0.973–3.045	0.062
2	2.457	1.210–4.991	**0.013**	3.949	1.806–8.633	**0.001**	2.525	1.385–4.606	**0.003**

UTUC: Upper tract urothelial carcinoma; LVI: Lymphovascular invasion.

### Development of new nomograms based on F-NLR and model performance

**Figure 3.**
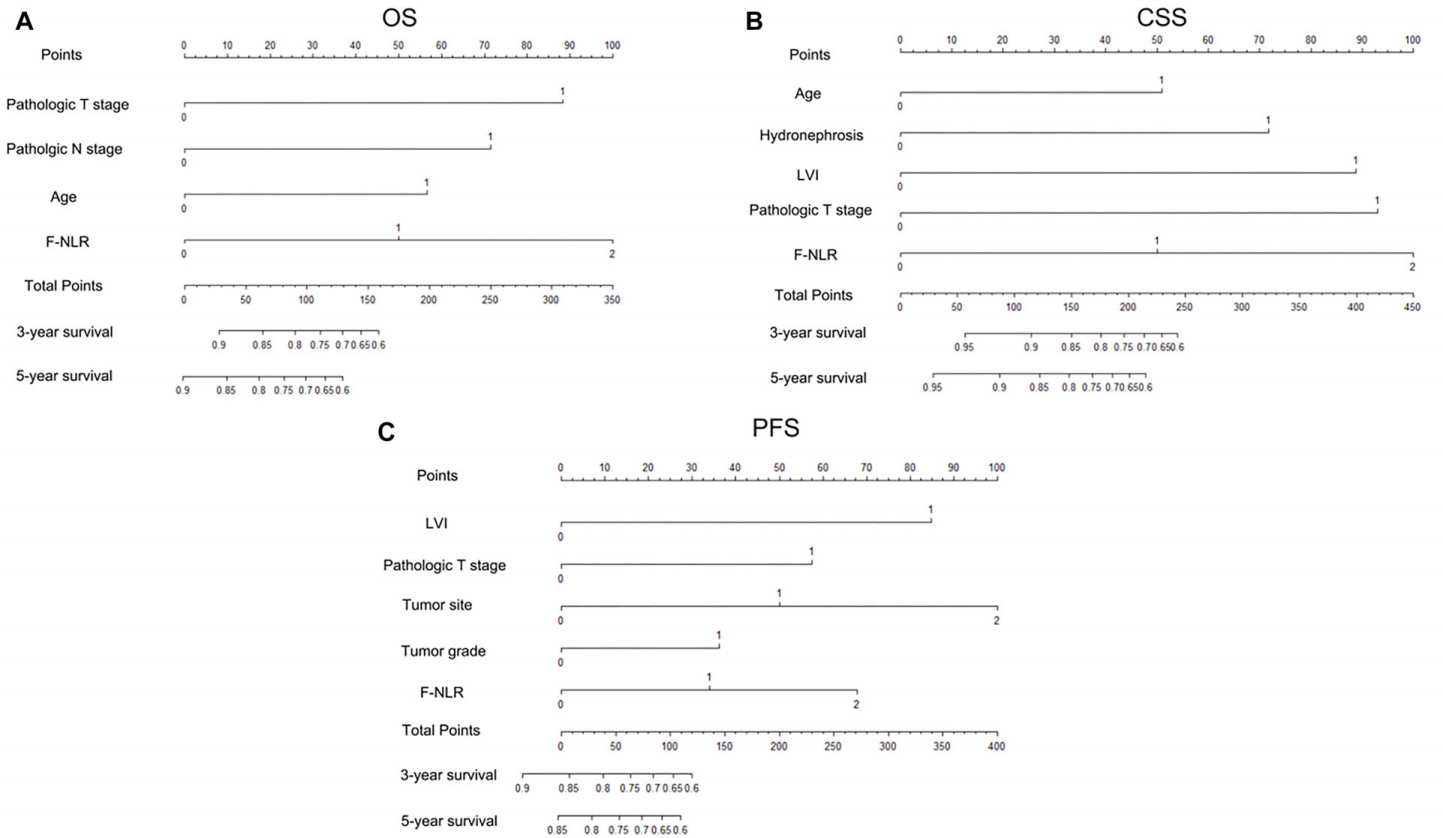
Established nomograms for OS (A), CSS (B), and PFS (C) in patients with UTUC. UTUC: Upper tract urothelial carcinoma; OS: Overall survival; CSS: Cancer-specific survival; PFS: Progression-free survival.

**Figure 4.**
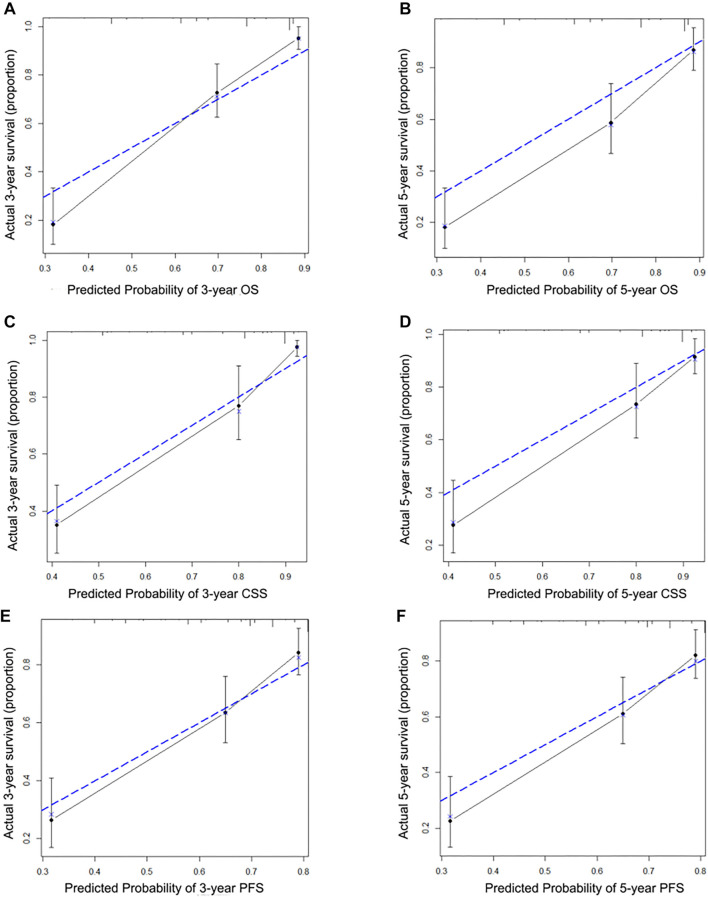
Calibration curve for predicting 3- and 5-year of OS (A and B), CSS (C and D), and PFS (E and F). OS: Overall survival; CSS: Cancer-specific survival; PFS: Progression-free survival.

**Figure 5.**
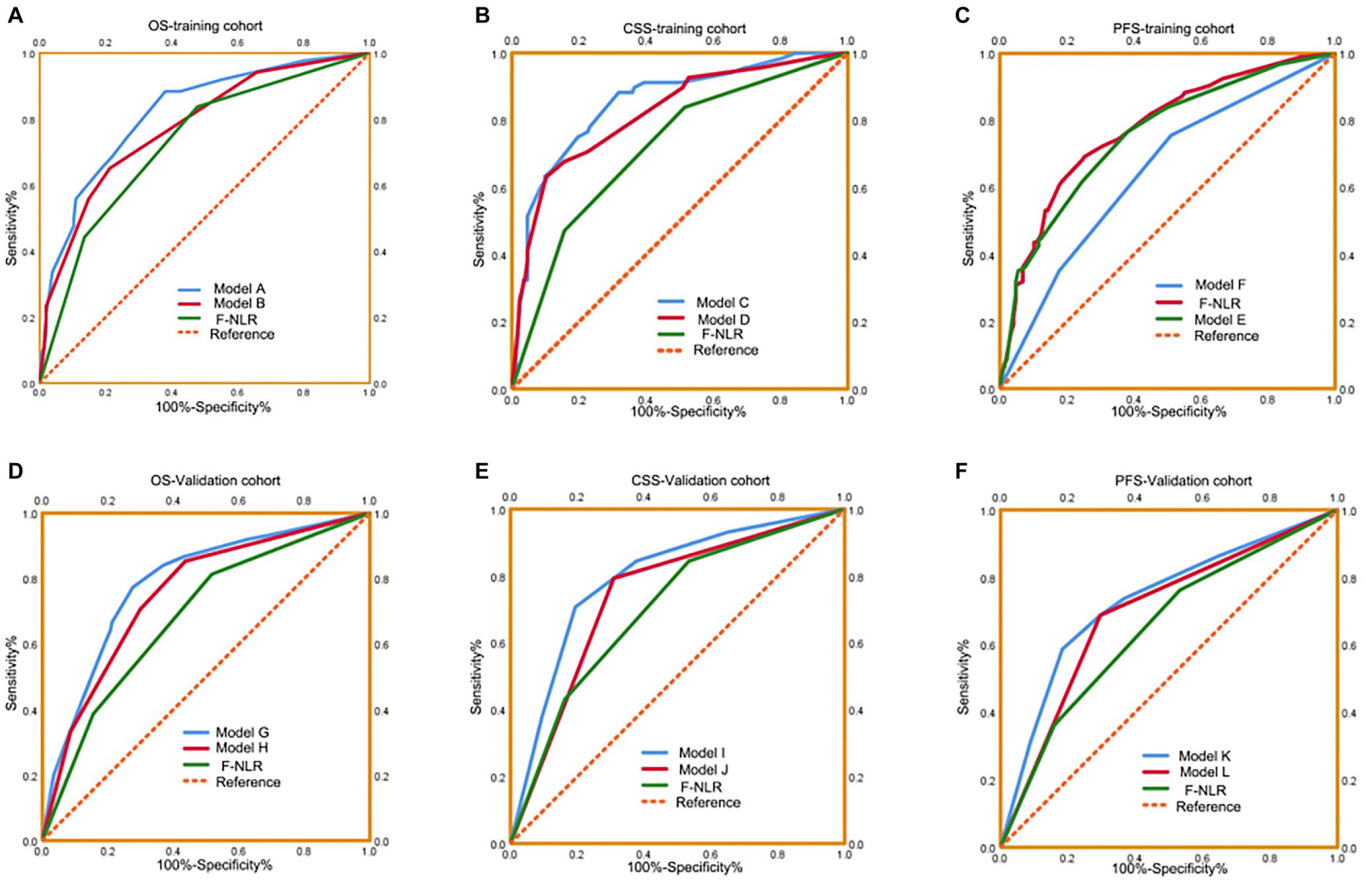
ROC analysis of the prognostic accuracy of F-NLR for OS, CSS, and PFS in established models in the training cohort (A–C) and validation cohort (D–F). OS: Overall survival; CSS: Cancer-specific survival; PFS: Progression-free survival; ROC: Receiver operating characteristic; F-NLR: Fibrinogen level and neutrophil-to-lymphocyte ratio.

Novel prognostic nomograms were developed based on independent variables to predict OS, CSS, and PFS at 3- and 5-year intervals (Figure 3). The C-index values for these nomograms were 0.808 for OS, 0.842 for CSS, and 0.727 for PFS, respectively. Calibration curves indicated a strong agreement between predicted and observed 3- and 5-year OS, CSS, and PFS probabilities (Figure 4). ROC curve analyses were also conducted to assess the clinical impact of F-NLR in both the training and validation cohorts. As shown in Figure 5 and Table 5, the AUC values of the models improved when F-NLR was included. These findings suggest that the new biomarker (F-NLR) has the potential to enhance prognostic accuracy for patients with UTUC.

**Table 5:** Predictive ability comparison of models for OS, CSS, and PFS with ROC analysis

**Model**	**AUC** **(95% CI)**	**Sensitivity** **(%)**	**Specificity** **(%)**	**Youden** **index**	**Positive likelihood ratio**	**Negative likelihood ratio**
*Training cohort*						
**For OS**						
Model A	0.818 (0.763–0.874)	88.37	38.06	0.503	2.322	0.306
Model B	0.778 (0.717–0.840)	65.12	78.71	0.438	3.058	0.443
F-NLR	0.729 (0.662–0.795)	83.72	52.25	0.360	3.261	0.312
**For CSS**						
Model C	0.853 (0.798–0.907)	88.24	68.21	0.564	2.775	0.172
Model D	0.821 (0.760–0.882)	63.24	89.60	0.528	6.078	0.410
F-NLR	0.718 (0.646–0.789)	83.83	48.56	0.324	1.629	0.333
**For PFS**						
Model E	0.774 (0.714–0.835)	69.15	74.83	0.440	2.747	0.412
Model F	0.755 (0.692–0.817)	76.60	61.90	0.385	2.011	0.378
F-NLR	0.645 (0.574–0.716)	75.53	48.98	0.245	1.480	0.500
*Validation cohort*						
**For OS**						
Model G	0.787 (0.725–0.849)	77.33	72.41	0.497	2.803	0.313
Model H	0.754 (0.689–0.820)	85.33	56.32	0.417	1.954	0.260
F-NLR	0.685 (0.614–0.756)	81.33	48.28	0.296	1.572	0.387
**For CSS**						
Model I	0.795 (0.729–0.861)	70.69	80.63	0.513	3.649	0.364
Model J	0.742 (0.670–0.814)	79.31	69.11	0.484	2.568	0.299
F-NLR	0.701 (0.625–0.776)	84.48	46.32	0.308	1.574	0.335
**For PFS**						
Model K	0.727 (0.657–0.798)	57.69	81.55	0.392	3.127	0.519
Model L	0.696 (0.625–0.767)	68.75	70.41	0.392	2.324	0.444
F-NLR	0.651 (0.577–0.724)	76.25	46.32	0.230	1.432	0.513

Model A: Age+pT+pN+F-NLR; Model B: Age+pT+pN; Model C: Age+Hydronephrosis+pT+LVI+F-NLR; Model D: Age+Hydronephrosis+pT+LVI; Model E: Tumor site+pT+Tumor grade+LVI+F-NLR; Model F: Tumor site+pT+Tumor grade+LVI; Model G: Anemia+pT+F-NLR; Model H: Anemia+pT; Model I: PT+ F-NLR; Model J: PT; Model K: PT+ F-NLR; Model L: pT. OS: Overall survival; CSS: Cancer-specific survival; PFS: Progression-free survival; ROC: Receiver operating characteristic.

## Discussion

Recently, an increasing number of studies have established integrative models that combine multiple clinicopathological parameters to more accurately predict oncological survival in patients with tumors [17, 18]. These studies suggest that the prognostic accuracy of these new models surpasses that of individual parameters. In the present study, the prognostic index F-NLR, which consists of three inflammation-coagulation indicators—neutrophils, lymphocytes, and fibrinogen—was evaluated. It was confirmed that F-NLR is associated with poor prognosis in UTUC. Patients were divided into three groups according to their F-NLR score (0, 1, or 2), and those with higher scores exhibited more aggressive clinicopathological characteristics. Furthermore, F-NLR was identified as an independent risk predictor, and nomograms based on F-NLR demonstrated strong predictive performance. Therefore, the F-NLR score could serve as a useful tool for accurately stratifying UTUC patients by risk.

Accumulating evidence suggests a close relationship between hyperfibrinogenemia and tumor progression [11, 19]. An earlier study reported a reduction in tumor metastasis in fibrinogen-deficient mice, concluding that fibrinogen plays a significant role in metastasis [20]. Fibrinogen, a key component of the coagulation cascade, can also be synthesized by cancer cells [21]. Two biological mechanisms may explain fibrinogen’s impact on tumor progression. First, fibrinogen promotes tumor progression by facilitating growth factors (such as vascular endothelial growth factor and fibroblast growth factor) in binding to receptors on the tumor cell surface [21]. Second, fibrinogen contributes to thrombosis by enhancing tumor cell adhesion to platelets, shielding cancer cells from natural killer cells [22]. Previous studies have found that hyperfibrinogenemia predicts worse outcomes in various cancers, including UTUC [15, 16]. In our study, high fibrinogen levels were associated with anemia, hypoproteinemia, advanced pathological T and N stages, and LVI. Additionally, high fibrinogen levels were linked to poorer OS, CSS, and PFS, suggesting that fibrinogen is a reliable and accessible biomarker for predicting post-surgical outcomes in UTUC patients.

The inflammatory response is widely recognized to influence tumor development and progression [23], largely due to the actions of circulating inflammatory cells, such as neutrophils and lymphocytes. Circulating lymphocytes, especially CD4+ T cells, play a vital role in immune defense against cancer cells. Lymphocytes exert antitumor effects by inducing cancer cell apoptosis and releasing cytokines like interferon (IFN)-γ and tumor necrosis factor (TNF)-α, which inhibit tumor growth and metastasis [24, 25]. Consequently, a reduction in lymphocyte count can weaken the immune response against tumors. Meanwhile, neutrophils may protect cancer cells from immune surveillance by inactivating T cells [26]. Tumor-associated neutrophils further support tumor growth, angiogenesis, and progression by releasing immunoregulatory mediators [27, 28].

The interaction between inflammation and coagulation cascades can facilitate tumor progression [16]. Treatments targeting fibrinogen reduction and inflammation modulation may improve cancer prognosis. F-NLR, an important blood marker that includes fibrinogen, neutrophils, and lymphocytes, has been associated with poorer oncological outcomes in various cancers [29]. Wang et al. [30] found that F-NLR levels significantly correlated with prognosis in non-small cell lung cancer patients undergoing radical surgery. Similarly, Li et al. [31] reported that F-NLR was a significant predictor of mortality in gastric cancer patients and that combining fibrinogen and NLR enhanced prognostic accuracy for this population.

In the present study, we categorized patients into three groups based on F-NLR scores (0, 1, and 2). A higher F-NLR score (≥2) was associated with adverse clinicopathological factors, such as anemia, hypoproteinemia, larger tumor size, advanced T and N stages, LVI, and an increased risk of recurrence and mortality. Patients with higher F-NLR scores had relatively shorter OS, CSS, and PFS than those with lower scores (0 or 1). The F-NLR score demonstrated valuable predictive capability for UTUC patients, enhancing the limited predictive power of fibrinogen, neutrophil, or lymphocyte levels alone. We also developed nomograms incorporating the F-NLR score and other significant independent factors, finding that models including F-NLR yielded higher AUC values. This simple and cost-effective marker can be used to identify high-risk UTUC patients in clinical practice, offering a practical alternative to tissue-based prognostic tools. However, the role of F-NLR in guiding treatment decisions and its prognostic value alongside other biomarkers warrant prospective validation in an independent cohort.

The study has several limitations. First, as a retrospective study, it may be subject to selection bias in patient enrollment and data collection. However, our departments (The First Affiliated Hospital of Wenzhou Medical University and the Third Clinical Institute Affiliated Hospital of Wenzhou Medical University) are the two largest urologic centers for UTUC in southern Zhejiang Province, providing a large sample size. The findings from our training cohort were also validated in an independent cohort, lending credibility to our data. Second, markers, such as IL-6 and C-reactive protein, which are also indicators of inflammation, were not included due to incomplete data. Third, patients with pre-existing metastasis at the time of RNU were excluded, limiting the generalizability of the results to all UTUC patients, particularly given the small sample size. We are planning a prospective study to further validate the impact of F-NLR across different UTUC subgroups, including patients with metastasis.

## Conclusion

A high F-NLR score has been identified as a significant risk factor for predicting OS, CSS, and PFS rates in UTUC patients after RNU. The authors hope that this reliable and economical tool can effectively stratify patients, guiding treatment strategies to improve patient outcomes.

## Supplemental data

**Table S1:** Baseline characteristics of patients with non-metastatic UTUC according to F-NLR score in validation cohorts

**Variables**	**F-NLR score**			***P* value**
	**0 (N ═ 95)**	**1 (N ═ 87)**	**2 (N ═ 59)**	
Age, years (>65/≤65)	53/45	62/33	33/23	0.285
Gender (male/female)	60/38	66/29	42/14	0.186
ASA grade (≥3/<3)	13/85	17/78	9/47	0.673
BMI, kg/m^2^ (≥25/<25)	36/62	29/66	17/39	0.589
Hydronephrosis (yes/no)	65/33	69/26	37/19	0.571
Hemoglobin, g/dL	127.36 ± 19.07	126.86 ± 17.91	119.77 ± 17.28	**0.031**
Anemia (yes/no)	19/79	32/63	24/32	**0.006**
Albumin, g/dL	40.72 ± 5.79	38.86 ± 3.34	37.64 ± 3.89	**<0.001**
Hypoproteinemia (yes/no)	3/95	8/87	11/45	**0.003**
Neutrophil, 10^9^/L	3.24 ± 1.01	5.20 ± 2.33	6.62 ± 4.23	**<0.001**
Lymphocytes, 10^9^/L	2.00 ± 0.52	1.47 ± 0.59	1.48 ± 0.68	**<0.001**
NLR	1.67 ± 0.47	4.12 ± 3.05	4.54 ± 1.93	**<0.001**
Fibrinogen, g/L	3.22 ± 0.64	3.58 ± 1.03	5.39 ± 0.86	**<0.001**
Tumor size, cm (≥3/<3)	28/70	31/64	28/28	**0.023**
Tumor site (pelvicalyceal/ureter/both)	54/41/3	51/38/6	32/18/3	0.347
Multifocality (yes/no)	19/79	26/69	13/46	0.423
Pathologic T stage (T1/T2/T3/T4)	43/28/23/4	34/23/34/4	6/10/29/11	**<0.001**
N stage (N1/N0)	1/97	5/90	9/47	**0.001**
High tumor grade (yes/no)	61/37	69/26	53/3	**<0.001**
LVI (yes/no)	4/94	16/79	15/41	**<0.001**
All-cause death, *n* (%)	14/84	32/63	29/27	**<0.001**
Cancer-specific mortality, *n* (%)	9/89	24/71	25/31	**<0.001**
Recurrence, *n* (%)	19/79	32/63	29/27	**<0.001**
Follow up duration, months, median (quartile)	54.65 ± 26.49	48.68 ± 26.59	34.55 ± 26.36	**<0.001**

UTUC: Upper tract urothelial carcinoma; LVI: Lymphovascular invasion.

## Data Availability

Some or all data used during the study are available from the corresponding author by request.
